# The Role of BDNF in Peripheral Nerve Regeneration: Activity-Dependent Treatments and Val66Met

**DOI:** 10.3389/fncel.2018.00522

**Published:** 2019-01-11

**Authors:** Claire Emma McGregor, Arthur W. English

**Affiliations:** Department of Cell Biology, Emory University School of Medicine, Atlanta, GA, United States

**Keywords:** peripheral nerve injury, BDNF, Val66Met, trkB, electrical stimulation, exercise, optogenetics

## Abstract

Despite the ability of peripheral nerves to spontaneously regenerate after injury, recovery is generally very poor. The neurotrophins have emerged as an important modulator of axon regeneration, particularly brain derived neurotrophic factor (BDNF). BDNF regulation and signaling, as well as its role in activity-dependent treatments including electrical stimulation, exercise, and optogenetic stimulation are discussed here. The importance of a single nucleotide polymorphism in the *BDNF* gene, Val66Met, which is present in 30% of the human population and may hinder the efficacy of these treatments in enhancing regeneration after injury is considered. Preliminary data are presented on the effectiveness of one such activity-dependent treatment, electrical stimulation, in enhancing axon regeneration in mice expressing the met allele of the Val66Met polymorphism.

## Peripheral Nerve Injury

Despite the ability of axons in peripheral nerves to regenerate, recovery is generally very poor (Portincasa et al., 2007; Scholz et al., 2009). The cellular changes that occur after an injury often cannot sustain axon regeneration for the duration required to reinnervate target organs (Fu and Gordon, 1995a,b). The neurotrophins have emerged as an important modulator of axon regeneration, particularly brain derived neurotrophic factor (BDNF). Here, we will review BDNF and its role in activity-dependent treatments to enhance regeneration. Then we will discuss a single nucleotide polymorphism in the *bdnf* gene, Val66Met, which is present in 30% of the human population and may hinder the efficacy of these treatments (Egan et al., 2003; Shimizu et al., 2004). Finally, we will present preliminary data on the effectiveness of one such activity-dependent treatment, electrical stimulation (ES), in enhancing axon regeneration in mice expressing the met allele of the Val66Met polymorphism.

## Brain Derived Neurotrophic Factor

BDNF is a member of the neurotrophin family, which also includes nerve growth factor (NGF), neurotrophin 3 (NT3), and neurotrophin 4/5 (NT4/5). BDNF is required for normal development—BDNF knockout (KO) is embryonic lethal (Jones et al., 1994; Schwartz et al., 1997). In adulthood, BDNF is involved in synaptic plasticity, long term potentiation (LTP), learning and memory as well as hippocampal neurogenesis and regeneration after injury (Lindsay, 1988; Lewin and Barde, 1996; Lu et al., 2014; Richner et al., 2014). In the subsequent paragraphs, we review how BDNF is regulated at the level of mRNA transcripts, protein trafficking, and receptor binding, following with its role in peripheral nerve regeneration.

### Regulation of BDNF Transcripts

**Figure 1:**
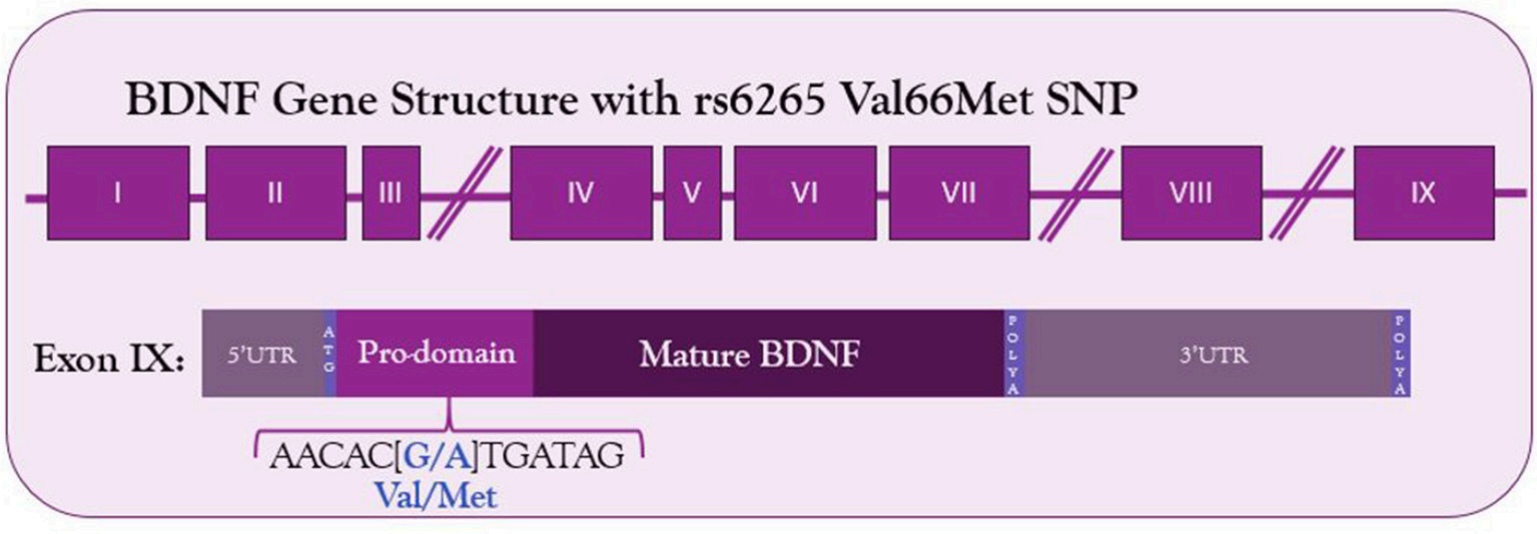
Structure of BDNF gene and location of Val66Met SNP in the coding exon IX. The G to A substitution in the prodomain results in a valine to methionine substitution and decreased Ca^2+^-dependent release of BDNF.

The human *BDNF* gene resides on the short arm of the 11th chromosome (Maisonpierre et al., 1991). It consists of 9 exons—eight 5′ untranslated exons and one protein coding 3′ exon (Figure 1) (Liu et al., 2006; Aid et al., 2007; Pruunsild et al., 2007). Through alternative splicing, 17 distinct mRNA transcripts for BDNF have been identified in humans and 11 in rodents (Pruunsild et al., 2007). Additionally, the 3′UTR of the gene contains two polyadenylation sites, resulting in both a long 3′UTR and a short 3′UTR, doubling the possible splice variants. The entire protein-coding region resides on exon IX, so the mature BDNF protein synthesized is identical regardless of mRNA splicing. Splice variants allow for spatial and temporal control of the BDNF transcript.

Spatial control of the 5′UTRs can be seen in exon expression throughout the body. BDNF transcripts containing exons I, II, and III are found exclusively in the brain, and transcripts containing exon IV are predominantly found peripherally in the lung and heart, but can also be found in brain tissue (Timmusk et al., 1993). Even within brain tissue, different promoters can be found in different cell types. For example, exon IV transcripts are required for proper GABAergic interneuron function in the prefrontal cortex (Sakata et al., 2009).

Many different stimuli exert temporal control over BDNF transcription. In cultured cortical neurons, Ca^2+^ influx results predominantly in transcription of exon IV-containing mRNA (Tao et al., 1998). This promoter contains a cAMP/Ca^2+^-response element-like element (CaRE3/CRE) that is required for activity-dependent transcription (Tao et al., 1998; Hong et al., 2008). The transcription factor CREB binds this element, is phosphorylated by calcium-regulated kinase cascades, and recruits transcriptional machinery resulting in Ca^2+^ dependent transcription of exon IV-containing BDNF mRNA (West et al., 2001; Lonze and Ginty, 2002). Other stimuli have been identified in modulating BDNF expression. In motoneurons, exon VI transcripts are androgen sensitive, despite no known androgen response element on the *bdnf* gene (Ottem et al., 2010; Sabatier and English, 2015). There is, however, an estrogen response element (Sohrabji et al., 1995). SRY-box containing gene 11 (Sox11), a transcription factor involved in neuronal survival, axon growth, and regeneration after injury, increases exon I containing BDNF mRNA transcripts specifically in peripheral DRG neurons, but not in CNS neurons (Jankowski et al., 2006; Salerno et al., 2012; Struebing et al., 2017). Exons II and VI are sensitive to tricyclic and atypical antidepressants (Vaghi et al., 2014).

A further role for 5′ promoter exons regulating BDNF mRNA may lie in mRNA trafficking. In both cortical and hippocampal neurons, BDNF mRNA is found in dendrites and activity induces trafficking of BDNF mRNA to distal dendrites (Tongiorgi et al., 1997; Capsoni et al., 1999; Chiaruttini et al., 2008, 2009). Interestingly, only certain splice variants are found in dendrites—those containing exons IIB, IIC, and VI (Pattabiraman et al., 2005; Chiaruttini et al., 2008). Transcripts containing exons I, III, and IV are restricted to the cell body.

Further spatial and temporal translational control of BDNF mRNA may come via the 3′UTR. The *bdnf* 3′ UTR contains two polyadenylation sites. This allows for both a long 3′UTR and a short 3′UTR to be transcribed (Timmusk et al., 1993; Aid et al., 2007; Pruunsild et al., 2007). These different 3′UTRs are thought to determine mRNA trafficking within the cell. Both the long and short 3′UTR transcripts can be found in the dendrites under different conditions (Vicario et al., 2015). However, in general, the long 3′UTR transcripts are trafficked to the dendrites, where local BDNF synthesis can regulate pruning and enlargement of synapses, whereas the short 3′UTR transcripts stay in the cell body (An et al., 2008). Both depolarization of the neuron as well as BDNF itself increase the number of BDNF mRNA transcripts targeted to the dendrites (Tongiorgi et al., 1997; Righi et al., 2000). Remarkably, the short 3′UTR transcripts account for the majority of BDNF translation, whereas the long 3′UTR transcripts are translationally repressed by RNA binding proteins which stabilize the mRNA until neural activity elicits rapid local translation (Lau et al., 2010; Allen et al., 2013; Vaghi et al., 2014). Calcium influx associated with neuronal activity also results in increased stabilization of BDNF mRNA (Fukuchi and Tsuda, 2010). Conversely, the 3′UTR also contains regions which interact with microRNAs, which are short, non-coding RNA strands that complement mRNA transcripts and result in transcript degradation (Bartel, 2004). Numerous microRNAs have been identified as regulators of BDNF, and microRNA access to binding sites depends on the presence of the long or short 3′UTR (Mellios et al., 2008; Varendi et al., 2014).

### BDNF Trafficking and Secretion

BDNF, like the other neurotrophins, is synthesized as a pre-proprotein. The pre-domain functions as a signaling peptide that directs synthesis to the endoplasmic reticulum (ER) for future packaging as a secretory protein. It is immediately cleaved to form proBDNF upon sequestration in the ER (Lessmann et al., 2003). Within the ER, proBDNF forms homodimers (Kolbeck et al., 1994). Both proBDNF and a further cleaved form, mature BDNF, can be packaged into vesicles and secreted. ProBDNF is approximately 29 kDa, and once cleaved, mature BDNF is approximately 14 kDa (Seidah et al., 1996a). Intracellularly, cleavage can occur within the trans-Golgi network or secretory vesicles by furin, a protease, and proprotein convertases PCSK6 and PC5-6b. Extracellularly, cleavage is executed by tissue plasminogen activator or matrix metalloproteinases, which are secreted in an activity-dependent manner (Krystosek and Seeds, 1981; Gualandris et al., 1996; Seidah et al., 1996a,b; Lee et al., 2001; Mowla et al., 2001; Hwang et al., 2005; Keifer et al., 2009; Nagappan et al., 2009; Yang et al., 2009). Once cleaved, the prodomain is not immediately degraded, and can be secreted with mature BDNF (Anastasia et al., 2013).

From the trans-Golgi network, BDNF is directed to two different secretory pathways: a constitutive pathway and a regulated, Ca^2+^ dependent pathway (Mowla et al., 1999; Lessmann et al., 2003; Kuczewski et al., 2009). The constitutive pathway consists of small granules (50–100 nm diameter) that fuse with the cell membrane near the neuronal somata and proximal processes. The regulated secretory pathway consists of larger granules (300 nm diameter) that fuse in distal processes and axon terminals (Conner et al., 1997; Kohara et al., 2001; Brigadski et al., 2005; Dieni et al., 2012). The dual pathway for release is distinctive of BDNF. The other neurotrophins are preferentially secreted through a constitutive pathway. Under normal conditions, most neuronal BDNF is packaged into the regulated pathway (Lu et al., 2014).

Two important interactions have been identified in the sorting of BDNF into the regulatory pathway. The first to be discovered was the interaction between the sorting receptor carboxipeptidase E (CPE) and a three-dimensional motif on the mature domain of BDNF (Lou et al., 2005). Knocking out CPE in cortical neurons blocks activity-dependent release of BDNF and increases constitutive release. Similarly, adding this motif to NGF redirected its release to the regulated secretory pathway (Lou et al., 2005). The second interaction is between the prodomain and the protein sortilin (Chen et al., 2005). Sortilin is localized predominantly to the Golgi apparatus and interacts with the BDNF prodomain to direct it to the regulated secretory pathway (Nielsen et al., 2001; Chen et al., 2005). When sortilin is unable to interact with the prodomain of BDNF, regulated release is decreased, but there is no compensatory increase in constitutive release (Chen et al., 2005; Lu et al., 2005). This has led to the hypothesis that the interaction between sortilin and the prodomain of BDNF is necessary for proper protein folding, which allows CPE to interact with the mature domain and sort BDNF into one of the two pathways (Lu et al., 2005). When NT-4/5, which is secreted constitutively, is modified to contain the BDNF prodomain, it is trafficked to the regulated secretory pathway (Brigadski et al., 2005). Similarly, blocking the cleavage of the prodomain of NGF, which is also released constitutively, results in its sorting into regulated secretory pathways (Mowla et al., 1999).

### BDNF Receptors

Once secreted, BDNF can bind to one of two receptors—tropomyosin receptor kinase B (trkB) or the common neurotrophin receptor, p75^NTR^. Mature BDNF preferentially binds trkB, resulting in pro-growth signaling, whereas proBDNF (as well as the other proneurotrophins) preferentially binds p75^NTR^, resulting in antigrowth signaling (Lee et al., 2001). BDNF is primarily secreted as proBDNF (Mowla et al., 1999, 2001; Chen et al., 2004). Thus the availability of proteins that cleave the prodomain may regulate which receptor is activated by BDNF release, providing another mechanism for control of BDNF signaling.

The trkB receptor is a typical tyrosine kinase. When ligand is bound, it dimerizes and autophosphorylates. In addition to BDNF, trkB can also bind NT-4/5. Several isoforms of trkB have been discovered, including isoforms that change its sensitivity to NT-4/5, as well as a truncated form that lacks an intracellular kinase domain (Eide et al., 1996). The truncated form acts as a dominant negative receptor, forming heterodimers with full length trkB receptors and blocking neurotrophin signaling (Eide et al., 1996; Fryer et al., 1997). Another possible role for truncated trkB on astrocytes and Schwann cells may be to act to control the pool of available neurotrophins, preventing them from degrading or signaling until released into the extracellular space (Alderson et al., 2000). In its full-length form, trkB has several intracellular tyrosine residues that can be phosphorylated (Huang and Reichardt, 2003). Three possible signaling cascades are then activated: phospholipase C gamma (PLCɤ); phosphotidyl-inositol-3 kinase (PI3K); and mitogen activated protein kinase/extracellular receptor kinase (MAPK/ERK) (Reichardt, 2006).

The phosphorylation of residue Y490 creates a binding site for adaptor protein Shc (Patapoutian and Reichardt, 2001). Shc binding trkB allows for activation of Ras and further activation of the MAPK/ERK pathway. Downstream of this pathway is mechanistic target of rapamycin (mTOR). Shc binding residue Y490 also results in the recruitment of PI3K and activation of protein kinase B (Akt) (Reichardt, 2006). The phosphorylation of residue Y785 creates a binding site for PLCɤ, which is then phosphorylated by trkB (Patapoutian and Reichardt, 2001). This phosphorylation activates PLCɤ, which then hydrolizes phosphotidylinositides to generate diacylglyerol (DAG), which activates protein kinase C (PKC), and inositol 1,4,5 triphosphate (IP_3_), which results in an influx of intracellular Ca2+ stores from the ER. These signaling cascades all converge at the level of the nucleus, where transcription is affected through CREB and other transcription factors (Minichiello, 2009).

Once bound to ligand, trkB is endocytosed to form a signaling endosome (Delcroix et al., 2003; Reichardt, 2006). Both ligand and receptor are contained within the endosome, allowing trkB to continue signaling as it is trafficked through the cell. In this way, trkB can be moved closer to the nucleus, where it can affect gene transcription, as well as brought into closer proximity to signaling effectors (Delcroix et al., 2003). However, not all actions of trkB happen at the level of the soma—BDNF-trkB activation has been shown to affect local protein synthesis in the growth cone as well (Yao et al., 2006).

The second receptor for BDNF is the pan-neurotrophin receptor, p75^NTR^ (Rodríguez-Tébar et al., 1992). Generally thought of as a pro-death receptor, p75^NTR^ is a member of the tumor necrosis factor receptor super family and contains a cytosolic death domain (Liepinsh et al., 1997; Locksley et al., 2001). It is expressed primarily during development, but sensory neurons and spinal motoneurons maintain low expression through adulthood (Ernfors et al., 1989; Heuer et al., 1990; Wyatt et al., 1990; Ibáñez and Simi, 2012). Its cytosolic domain is non-enzymatic, so its actions depend entirely on associations with cytoplasmic proteins (Nagata, 1997). Despite its canonical role, p75^NTR^ can mediate both pro-death and pro-survival signals depending on its cytosolic partners. For example, p75^NTR^ is required during development for normal neuron growth and ramification (Yamashita et al., 1999). Multiple adaptor complexes interact with its cytosolic domain to mediate downstream effects (Dechant and Barde, 2002).

Additionally, p75^NTR^ has multiple membrane-bound and extracellular binding partners which can alter whether its signaling is pro-survival or pro-death. Through extracellular pairing with sortilin, p75^NTR^ is able to bind the proneurotrophins (Nykjaer et al., 2004; Teng et al., 2005), resulting in pro-death or anti-growth signaling through downstream JNK activation or caspase activation (Reichardt, 2006). However, neurotrophin binding to p75^NTR^ can also result in NFκB activity, which is a pro-survival signal (Carter et al., 1996; Hamanoue et al., 1999; Middleton et al., 2000). One key protein regulated by p75^NTR^ is RhoA, a small GTPase that regulates the actin cytoskeleton and inhibits axon elongation (Walsh et al., 1999; Schmidt and Hall, 2002). Through such interactions with the so-called death domain of p75^NTR^, neurotrophin binding inhibits Rho (Yamashita et al., 1999; Roux and Barker, 2002). Through forming a receptor complex with the Nogo receptor, NgR1, p75^NTR^ can act as a receptor for myelin-associated glycoprotein (MAG) (Wang et al., 2002), which enhances Rho activation and results in neurite collapse (Mi et al., 2004). Curiously, p75^NTR^ can act as a binding partner for the trks, including trkB, and increases affinity and selectivity of binding and thus enhancing trk signaling (Bibel et al., 1999).

Like trkB, truncated forms of p75^NTR^ have been identified. One short p75^NTR^ isoform lacks an extracellular ligand binding domain, but contains its intracellular machinery (Roux and Barker, 2002). This form is unable to bind the neurotrophins (Dechant and Barde, 1997). The extracellular domain of p75^NTR^ can also be cleaved by extracellular metalloproteinases (Roux and Barker, 2002). These isoforms could act as modulators of neurotrophin signaling.

The two receptors for BDNF are generally thought to have opposing roles and may mediate a balance between growth and death. trkB has a higher affinity for mBDNF, but as levels of neurotrophin increase, p75^NTR^ will also bind mBDNF and activate signals in direct opposition to trkB. Because of the different affinities for pro- and mature BDNF, cleavage of BDNF becomes another mechanism to control its downstream signaling effects (Lee et al., 2001). Depolarization of a neuron, which results in secretion of BDNF, also results in secretion of tissue plasminogen activator which cleaves proBDNF to create mature BDNF (Gualandris et al., 1996). BDNF-trkB signaling increases expression of matrix metalloproteinase 9, which also cleaves BDNF (Kuzniewska et al., 2013).

## Role of BDNF in Peripheral Nerve Injury

In peripheral nerves, BDNF is synthesized by motoneurons, a subset of DRG neurons, and Schwann cells (Apfel et al., 1996; Cho et al., 1997; Michael et al., 1997). After nerve crush or complete transection, BDNF mRNA increases in all three cell types, including in trkB- and trkC-expressing DRG neurons not found previously to express BDNF (Meyer et al., 1992; Funakoshi et al., 1993; Kobayashi et al., 1996; Michael et al., 1999; Al Majed et al., 2000a; English et al., 2007). BDNF mRNA can be found in low levels in the sciatic nerve, and after injury, that expression is upregulated. This upregulation is sustained over the course of weeks and can be attributed to both neuronal and non-neuronal sources (Meyer et al., 1992; Funakoshi et al., 1993). In facial nerve injury, upregulation of BDNF is correlated with enhanced functional outcome (Grosheva et al., 2016).

Following sciatic nerve injury, a transient increase in both BDNF and full length trkB mRNA is found in motoneurons (Kobayashi et al., 1996; Al Majed et al., 2000a). Unlike sensory neurons, NGF and trkA are not expressed by motoneurons, nor are they upregulated after injury (Funakoshi et al., 1993; Escandon et al., 1994). There is a small and short-lived upregulation of NT3 and NT4/5 in motoneurons (Funakoshi et al., 1993). TrkC is expressed by adult motoneurons, but it is not upregulated after injury (Johnson et al., 1999). Thus, the rapid upregulation of BDNF and trkB make it likely that BDNF is the main neurotrophin mediating early motoneuron response to nerve injury (Boyd and Gordon, 2003).

Schwann cells express only the truncated form of trkB, which has the potential to act as a dominant negative receptor for BDNF and NT4/5. Schwann cell truncated trkB mRNA levels decrease significantly after sciatic nerve injury (Frisén et al., 1993). This could be viewed as pro-regenerative, enabling available BDNF to bind to trkB receptors on regenerating neurites and enhance their growth. Conversely, after injury, Schwann cells upregulate p75^NTR^, which has been suggested to result in sequestration of neurotrophins and inhibit regeneration (Taniuchi et al., 1986; Bibel et al., 1999; Scott and Ramer, 2010).

### trkB in Peripheral Nerve Injury

Axons regenerate through the formation of growth cones, which need cytoskeletal proteins, such as actin and tubulin, to extend and stabilize the new growth. Beta actin mRNA is localized to peripheral axons, and peripheral nerve injury triggers actin mRNA to be transported down the axon for local protein synthesis (Koenig et al., 2000; Sotelo-Silveira et al., 2008; Willis et al., 2011). BDNF/trkB signaling triggers local translation of transported mRNAs through a Ca^2+^-dependent mechanism, and this is required for bidirectional turning toward BDNF (Yao et al., 2006). BDNF application to injured axons increases the number of actin waves (transport of actin filaments and associated proteins toward the growth cone) per hour (Difato et al., 2011; Inagaki and Katsuno, 2017). Neurotrophins also stimulate growth cone sprouting and actin accumulation in the sprouts (Gallo and Letourneau, 1998). Both of these processes are mediated through the PIP3/PI3K signaling pathway described above (Asano et al., 2008). When an actin wave reaches the growth cone, the growth cone enlarges, branches, and undergoes forward expansion (Flynn et al., 2009). Application of BDNF to growth cones results in microtubule reorganization to form lamellipodial as well as filopodial elongation (Gibney and Zheng, 2003).

In addition to local protein synthesis, trkB signaling has effects on cyclic AMP (cAMP) production, which may be important for the initial extension of growth cones across the site of injury. The MAPK/ERK pathway of BDNF/trkB signaling results in inhibition of phosphodiesterases (PDE) which normally degrade cAMP (Gao et al., 2003). As such, BDNF/trkB signaling results in increased levels of cAMP (Souness et al., 2000; Gao et al., 2003). This pathway has been shown to be necessary to overcome inhibition by MAG, and therefore PDE inhibition has been most thoroughly studied in models of spinal cord injury, where MAG inhibition of axon growth creates a substantial barrier to regeneration (Cai et al., 1999; Gao et al., 2003; Batty et al., 2017). Although injured peripheral nerves do not suffer inhibition by MAG to the same extent as that seen in the central nervous system, early in the regeneration process, inhibitory proteoglycans and myelin debris form an impermissible environment for axon regeneration (shen et al., 1998). Increasing cAMP through PDE inhibition enhances peripheral regeneration after injury, and it is likely that trkB activation contributes to this cAMP-mediated effect on regeneration (Gordon et al., 2009; Udina et al., 2010).

The different neurotrophin signaling pathways activated through trk receptors converge at the level of transcription in the nucleus. CREB, resulting from trkB-generated PI3K-Akt activation, increases sensory neurite outgrowth (White et al., 2000). Inhibiting phosphatase and tensin homolog (PTEN), an endogenous inhibitor of the PI3K pathway, through genetic knock out or pharmacology, enhances peripheral nerve regeneration *in vivo* and neurite outgrowth *in vitro* (Park et al., 2008; Christie et al., 2010). Numerous other transcription factors downstream of MAPK/ERK signaling, such as c-jun, STAT3, and ATF-3, have all been associated with changes in gene expression after injury that enhance survival and regeneration (Makwana and Raivich, 2005). mTOR, also downstream of MAPK/ERK signaling and repressed by PTEN, regulates protein synthesis and is also beneficial for DRG regeneration after injury (Park et al., 2008; Abe et al., 2010).

### p75^NTR^ in Peripheral Nerve Injury

Because of its roles in both pro-death and pro-survival signaling, it is not surprising that the role of p75^NTR^ in regeneration after injury has been controversial. Although generally considered an anti-growth signal, its role is far more complex as evidenced by conflicting results using p75^NTR^ knock-out mice.

Although expression is high during development, mature Schwann cells do not express p75^NTR^. Schwann cell expression of p75^NTR^ increases after injury (Taniuchi et al., 1986; Heumann et al., 1987a,b). Deletion of p75^NTR^ in Schwann cells mediates improved regeneration in DRG neurons (Scott and Ramer, 2010). Conversely, for motoneurons, Schwann cell p75^NTR^ deletion results in diminished functional recovery and axonal growth (Tomita et al., 2007). Expression of p75^NTR^ is thought to mediate remyelination through a BDNF-dependent mechanism. Disruption of endogenous BDNF signaling impairs myelination (Cosgaya et al., 2002; Zhang et al., 2008), as does p75^NTR^ knockout from Schwann cells (Cosgaya et al., 2002; Song et al., 2006; Tomita et al., 2007).

In motoneurons, p75^NTR^ levels rise dramatically after injury, returning to baseline levels by 30 days (Raivich and Kreutzberg, 1987; Yan and Johnson, 1988; Ernfors et al., 1989; Koliatsos et al., 1991; Saika et al., 1991; Rende et al., 1995; Gschwendtner et al., 2003). This upregulation in p75^NTR^ does not result in motoneuron cell death, however (Bueker and Meyers, 1951; Kuzis et al., 1999). Treating injured motoneurons with low-levels of recombinant human BDNF enhances their regeneration. Higher doses, however, result in failure to regenerate, which can be reversed by p75^NTR^ blockade (Boyd and Gordon, 2002). There is currently no motoneuron-specific p75^NTR^ knockout model, but conflicting results have been found with regard to motoneuron regeneration in p75^NTR^ pan-knockout mice. Boyd and Gordon found improved motor axon regeneration in knockout mice after peroneal nerve transection (Boyd and Gordon, 2001). Gschwendtner et al. found no effect of knocking out p75^NTR^ on facial nerve axon regeneration (Gschwendtner et al., 2003). Ferri et al. found worse axon regeneration but improved functional recovery after facial nerve crush (Ferri et al., 1998). Song et al. found fewer regenerating axons in p75^NTR^ knockout mice using both sciatic nerve and facial nerve crush injuries (Song et al., 2009). Most recently, Zhang et al. found worse axonal regeneration among p75^NTR^ knockout mice using a facial nerve crush model (Zhang et al., 2010). Using cell-type specific knockout of p75^NTR^ or targeting the binding partners of p75^NTR^ that result in different signaling could provide clarity to these conflicting results in how p75^NTR^ is affecting motoneuron regeneration.

Sensory neurons decrease expression of p75^NTR^ after injury (Zhou et al., 1996). Unlike adult motoneurons, there is significant cell death of DRG neurons after an injury, but this is restricted to small-diameter, mainly cutaneous afferent neurons (Welin et al., 2008; Wiberg et al., 2018). Cell death in these smaller DRG neurons can be blocked by application of NGF (Rich et al., 1987; Ljungberg et al., 1999). Likewise, in injured DRG neurons, BDNF acts in an autocrine loop to prevent cell death, and disruption in BDNF expression increases cell death (Acheson et al., 1995). A decrease in expression of p75^NTR^ could act as a survival signal, such that the endogenous increased neurotrophin secretion would be more likely to bind trk receptors and result in pro-survival signaling. In support of this hypothesis, in cultures of DRG neurons in which p75^NTR^ has been rendered inactive, cell survival is higher, and increased neurotrophin concentration does not result in increased cell death (Zhou et al., 2005). Similarly, disrupting the NT binding domain of p75^NTR^ results in increased sprouting after injury in DRG neurons (Scott et al., 2005).

Despite the upregulation of BDNF and its receptors after injury, neither BDNF nor the other neurotrophins (NGF, NT3, and NT4/5), is required for spontaneous regeneration of peripheral neurons (Diamond et al., 1987, 1992; Streppel et al., 2002; Wilhelm et al., 2012). However, application of exogenous BDNF enhances axonal regeneration, functional recovery and decreases synaptic stripping (Lewin et al., 1997; Boyd and Gordon, 2002, 2003; Davis-Lopez de Carrizosa et al., 2009). Recently, small molecule trkB agonists have been developed, and these also enhance regeneration after injury (English et al., 2013).

## Activity Dependent Treatments Enhance Regeneration

The first published report using activity-dependent treatments to enhance peripheral nerve regeneration was from Hines in 1942. He tested both electrical stimulation (ES) and different exercise paradigms in enhancing functional outcome in rats with tibial nerve transections (Hines, 1942). Since then, there has been great interest in treatments which activate injured neurons, collectively known as activity-dependent treatments, to enhance nerve regeneration (Udina et al., 2011a). These treatments include ES (Table 1), exercise (Tables 2–4), and more recently, optogenetic stimulation (Table 5). One benefit of activity-dependent treatments is the potential for easy translation from bench to bedside—using ES and exercise in human patients would require meeting far fewer regulatory requirements than the use of an experimental drug. Moreover, for nerve injuries that require surgical intervention, ES could be performed easily at the time of surgical repair of the nerve, as has already begun with clinical trials for patients undergoing surgery for carpal tunnel release and complete digital nerve transection (Gordon et al., 2010; Wong et al., 2015). Exercise has the advantage of being low cost and allowing patients to take control of their recovery. However, in the case of extensive injuries, exercise may not be an option for a recovering patient. For this reason, finding treatments that mimic the effects of exercise, such as optogenetic stimulation, may be advantageous in treating patients. To accomplish such a goal, an understanding of the biological basis for the enhancement seen with these treatments is necessary.

**Table 1:** Effect of electrical stimulation on peripheral nerve regeneration.

**Regimen**		**Model**	**Result**		**References**
Unspecified	3 min/day	Rat tibial nerve crush	Functional recovery	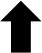	Hines, 1942
4 Hz	24 h/4 weeks	Rabbit soleus nerve crush	Functional recovery	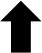	Nix and Hopf, 1983
20 hz	15–60 min	Rat sciatic nerve crush	Functional recovery	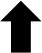	Pockett and Gavin, 1985
20 Hz	60 min, 24 h, 1 week, 2 weeks	Rat femoral nerve transection	Axonal growth	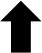	Al Majed et al., 2000b
20 Hz	8 h/day/4 weeks	Rat sciatic nerve avulsion	Functional Recovery	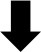	Tam et al., 2001
100 hz	10 pulses/2 min	Cultured rat retinal ganglion cell	Neurite outgrowth		Goldberg et al., 2002
20 Hz	1 h	Rat femoral nerve transection	Axonal growth	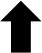	Brushart et al., 2002
20 Hz	1 h	Rat femoral nerve transection	Axonal growth	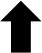	Brushart et al., 2005
20 Hz	1 h	Thy1-H-YFP mouse fibular nerve transection	Axonal growth	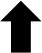	English et al., 2007
20 Hz	1 h, 3 h, 24 h, 1 week, 2 weeks	Rat femoral nerve transection	1hr-Axonal growth Others—no change	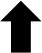	Geremia et al., 2007
20 Hz	1 h	Mouse femoral nerve transection	Functional recovery	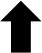	Ahlborn et al., 2007
?	30 min/day until recovery	Rat facial nerve transection	Functional recovery	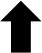	Lal et al., 2008
20 Hz	1 h	Rat sciatic nerve transection	Functional Recovery Axonal growth	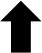	Vivo et al., 2008
20 hz	1 h	Human carpal tunnel syndrome release surgery	Functional recovery	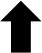	Gordon et al., 2010
20 Hz	3 days	Rat adult cultured DRG neurons	Neurite outgrowth	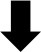	Enes et al., 2010
20 Hz	1 h	Rat sciatic nerve crush	Myelination and myelin thickness	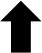	Wan et al., 2010
20 Hz	1 h	Thy1-H-YFP mouse sciatic nerve transection	Axonal growth	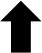	Singh et al., 2012
20 Hz	1 h	Rat cultured DRG neurons	Neurite outgrowth		Singh et al., 2012
20 Hz	30 min/day 1–7days	Rat facial nerve crush	Functional recovery	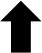	Foecking et al., 2012
20 Hz	20 min	Rat sciatic nerve Delayed repair 2 h−24 weeks	Axonal growth Functional recovery	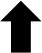	Huang et al., 2013
20 Hz	1 h	Human digital nerve transection	Functional sensory recovery	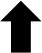	Wong et al., 2015
20 Hz	1 h	Rat common peroneal nerve transection Delayed repair 3 months	Axonal growth	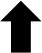	Elzinga et al., 2015

*Regimen specifies stimulation paradigm. Model specifies which animal and injury model was used. Result specifies what outcome measure was analyzed. 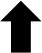 Denotes improvement in outcome measured, 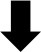 denotes worse outcome*.

**Table 2:** Effects of treadmill training on peripheral nerve regeneration.

**Regimen**	**Duration**	**Delay**	**Model**	**Result**		**References**
6.5–27 m/min 10–40 min/day	1-2/day	2-3 weeks	Rat sciatic nerve crush ♀	Functional recovery	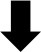	Herbison et al., 1980a
27 m/min 1–2/day 5 days/week	3–4 weeks	2–3 weeks	Rat sciatic nerve crush ♀	No change		Herbison et al., 1980b
1 h/day 26.8 m/min	10 weeks	Prior to injury	Rat L4 root transection ♀	Increased sprouting		Gardiner et al., 1984
10 m/min 30 min/twice/day	21 days	None	Rat sciatic nerve crush ♂	Functional recovery	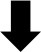	van Meeteren et al., 1998
10 m/min 1.5 h/twice/day 5 days/week	10 weeks	1 week	Rat peroneal nerve transection ♀	Functional recovery	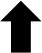	Marqueste et al., 2004
18 m/min 30 min/twice/day	2 weeks	12 h	Rat sciatic nerve crush ♂	Axonal growth	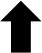	Seo et al., 2006
10 m/min*1 h/day 20 m/min Or 2 min*4/day 5 days/week	2 weeks	3 days	Thy1-H-YFP mouse sciatic nerve transection	Axonal growth	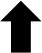	Sabatier et al., 2008
8 m/min 30 min/twice/day	2 weeks	12 h	Rat sciatic nerve crush ♂ DRG culture ♂	Axonal growth Neurite length	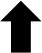	Seo et al., 2009
10 m/min*1 h/day 20 m/min 2 min*4/day 5 days/week	2 weeks	3 days	Mouse sciatic nerve transection	Axonal growth	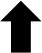	English et al., 2009
20 Hz 1 h ES + 5 m/min 2h/day	4 weeks	5 days	Rat sciatic nerve transection ♀	Axonal growth	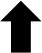	Asensio-Pinilla et al., 2009
20 cm/s-54 cm/s 60 min/day 5 days/week	5 or 52 days	3 days	Mouse sciatic nerve chronic constriction injury ♂	Functional recovery	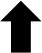	Cobianchi et al., 2010
4.6 m/min 30min/twice/day	4 weeks	5 days	Rat sciatic nerve transection ♀	Functional Recovery Axonal growth	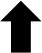	Udina et al., 2011b
1.8–3 m/min 20 min/day	3 weeks	7 days	Rat ulnar nerve crush ♂	Functional recovery	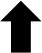	Pagnussat et al., 2012
10 m/min 1 h/5 days/week	2 weeks	3 days	Rat sciatic nerve transection ♀	Functional recovery	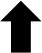	Boeltz et al., 2013
10 m/min 1 h/5 days/week	2 weeks	3 days	Mouse sciatic nerve transection ♀ ♂	Synaptic stripping	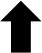	Liu et al., 2014
10 m/min 1 h/5 days/week	6 weeks	3 days	Mouse median nerve transection ♂	Functional recovery	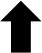	Park and Höke, 2014
20 m/min 2 min*4/day 5 days/week	2 weeks	3 days	SLICK::BDNF^f/f^ mouse sciatic nerve transection ♀	Synaptic stripping	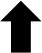	Krakowiak et al., 2015

*Regimen is the speed of running and for how long each day. Duration is how many days TT was performed. Delay refers to how long after injury before exercise was performed. Model specifies what type of injury and in what animal. Sex of animals is specified by ♀ or ♂. If this is not listed, it was not specified. Result specifies what outcome measure was analyzed. 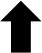
Denotes improvement in outcome measured, 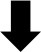 denotes worse outcome. No arrow denotes no effect*.

**Table 3:** Effect of swimming exercise on peripheral nerve regeneration.

**Regimen**	**Delay**	**Model**	**Result**		**References**
1 h/day	Unspecified	Rat tibial nerve transection	Functional recovery	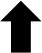	Hines, 1942
10 min/day 10 days	4, 11, 18 days	Rabbit sciatic nerve crush	Myelination	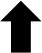	Sarikcioglu and Oguz, 2001
30 min/day 2 weeks	None 2 weeks	Rat sciatic nerve crush ♂	Decreased sprouting		Teodori et al., 2011
10–30 min/day 3 days/week 3 weeks	7 days	Rat sciatic nerve transection	No change		Liao et al., 2017

*Regimen specifies how long swimming exercise lasted each day and how many days swimming was performed. Delay refers to how long after injury before exercise was performed. Model specifies what type of injury and in what animal. Sex of animals is specified by ♀ or ♂. If this is not listed, it was not specified. Result specifies what outcome measure was analyzed. 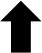 Denotes improvement in outcome measured, 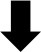 denotes worse outcome. No arrow denotes no effect*.

**Table 4:** Effect of other exercise paradigms on peripheral nerve regeneration.

**Exercise**	**Regimen**	**Delay**	**Model**	**Result**		**References**
Forced Wheel Running	2 h/day	Unspecified	Rat tibial nerve transection	Functional Recovery	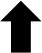	Hines, 1942
Overwork	Chronic	None	Rat sciatic nerve crush ♀	Functional recovery	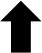	Herbison et al., 1973
Voluntary wheel running	4 weeks	None	Mouse tibial nerve transection	Functional recovery Axonal growth	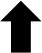	Badke et al., 1989
				Axonal growth	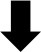	
Stretch Training	24 days	None	Rat sciatic nerve crush ♀	Functional recovery	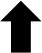	van Meeteren et al., 1997
Voluntary wheel running	8 h/day	None	L4 and L5 avulsion ♀	Axonal growth Functional Recovery	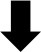	Tam et al., 2001
				Functional Recovery	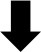	
Voluntary wheel running	3 or 7 days	Prior to injury	Rat DRG culture Rat sciatic nerve crush	Neurite outgrowth Axonal growth	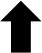	Molteni et al., 2004
Manual Whisker Stimulation	5 min/day 5 days/week 2 months	1 day	Rat facial nerve transection ♀	Functional Recovery	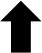	Angelov et al., 2007
Manual Muscle Stimulation	5 min/day 5 days/week 2 months	1 day	Rat hypoglossal transection ♀	Functional Recovery	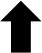	Guntinas-Lichius et al., 2007
Manual Whisker Stimulation	5 min/day 5 days/week 2 months	1 day	Rat hypoglossal transection ♀	Functional Recovery	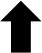	Evgenieva et al., 2008
Manual Muscle Stimulation	5 min/day 5 days/week 2 months	1 day	Rat facial nerve transection ♀	Functional Recovery	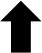	Bischoff et al., 2009
Manual Whisker Stimulation	5 min/day 5 days/week 2 months	1 day	BDNF^+/−^ or trkB^+/−^ rat facial nerve transection ♀	Functional Recovery	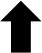	Sohnchen et al., 2010
Passive bicycle training	45 rpm 30 min/twice/day 4 weeks	5 days	Rat sciatic nerve transection ♀	Functional Recovery Axonal growth	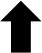	Udina et al., 2011b
Skilled Motor Task	20 min/day 3 weeks	7 days	Rat ulnar nerve crush ♂	Functional recovery	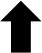	Pagnussat et al., 2012

*Exercise refers to what type of paradigm was used. Regimen specifies how long exercise lasted each day and how many days exercise was performed. Delay refers to how long after injury before exercise was performed. Model specifies what type of injury and in what animal. Sex of animals is specified by ♀ or ♂. If this is not listed, it was not specified. Result specifies what outcome measure was analyzed. 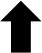 Denotes improvement in outcome measured, 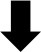 denotes worse outcome. No arrow denotes no effect*.

**Table 5:** Effect of optogenetic stimulation on peripheral nerve regeneration.

**Regimen**	**Mouse**	**Model**	**Result**		**References**
1 h 20 Hz 5 ms pulse	Thy1ChR2	Neonate DRG explant	Neurite outgrowth	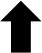	Park et al., 2015
1 h 20 Hz 1 ms pulse	Thy1ChR2	Sciatic nerve transection	Axonal outgrowth	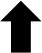	Ward et al., 2016
1–2 h 10–20 Hz (72 k pulse total) 1 ms pulse	Avil-Cre::ChR2-YFP^f/f^ Chat-ChR2-YFP	Sciatic nerve transection	Axonal outgrowth	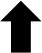	Ward et al., 2018

*Regimen specifies stimulation paradigm. Mouse specifies what transgenic mouse model was used. Result specifies what outcome measure was analyzed. 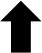 Denotes improvement in outcome measured, 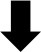 denotes worse outcome*.

### Electrical Stimulation

Immediately after a peripheral nerve injury, a calcium wave propagates along the cut axons toward their cell bodies. Blocking this calcium wave through inhibition of voltage gated calcium channels or inhibition of calcium release from the neuronal endoplasmic reticulum blocks regeneration (Ghosh-Roy et al., 2010). It has been proposed that ES mimics the retrograde calcium wave that propagates at the time of injury in order to elicit cell-autonomous mechanisms that initiate regeneration (Mar et al., 2014). This hypothesis is supported by evidence that ES enhances early regeneration by accelerating the process of axons crossing the site of injury to enter endoneurial tubes in the segment of the nerve distal to the injury (Brushart et al., 2002). ES results in a doubling of the number of motoneurons crossing the site of injury into the distal nerve at 1 week after nerve injury (Brushart et al., 2002). Without treatment, axons can take as long as 4 weeks to cross the site of injury, but by 3 weeks after injury, Al Majed et al. found all electrically stimulated motoneurons had already regenerated to their target muscle compared to 8 weeks for untreated controls (Al Majed et al., 2000b; Brushart et al., 2002).

The first applications of ES focused on the functional recovery of the affected muscles. In 1983; Nix and Hopf described that as early as 2 weeks after injury, treatment with 4 Hz stimulation 24 h daily increased twitch force, tetanic tension, and muscle action potentials (Nix and Hopf, 1983). In 1985; Pockett and Gavin found earlier return of the plantar extensor reflex with just 15–60 min of 20 Hz stimulation (Pockett and Gavin, 1985). Al Majed et al. chose their 20Hz regimen based on the mean physiological frequency of motoneuron discharge and tried numerous stimulation regimens, stimulating continuously for 1 h, 1 day, 1 week, and 2 weeks. They were the first to examine the effect of ES on the regenerating axons (Loeb et al., 1987; Al Majed et al., 2000b). Just 1 h of 20 Hz stimulation resulted in long-lasting enhancement of peripheral nerve regeneration. Following publication of this paper, 20 Hz stimulation became the standard for studying ES (Table 1).

Without treatment, axon regeneration into motor or sensory pathways in the distal segment of a cut nerve is random for the first 2 weeks following injury (Brushart, 1993). Motoneurons whose axons have entered only sensory pathways (endoneurial tubes previously occupied by cutaneous axons) remain permanently mistargeted (Brushart, 1993). Enhancing the speed of regeneration but increasing mistargeting could result in poorer functional recovery. However, ES has been shown to increase the sensorimotor specificity of regenerating axons after peripheral nerve injury. More motoneurons regenerate exclusively into motor pathways in rats treated with ES (Al Majed et al., 2000b). Fewer than 40% of injured DRG neurons reinnervated sensory pathways in controls compared to 75% in ES-treated animals (Brushart et al., 2005). However, innervating a motor endoneurial tube does not necessitate reaching the appropriate muscle target. Indeed, topographic analysis of motoneuron regeneration after ES revealed *increased* misdirection of regenerating motor axons to functionally inappropriate targets by over 500% (English, 2005). This misdirection resulted in motoneurons previously innervating extensor muscles reinnervating antagonistic flexor muscles.

Appropriate pathway innervation may rely on Schwann cells secreting specific growth factors for motor and sensory tubes. Schwann cells in the cutaneous nerves express high levels of NGF after injury, whereas Schwann cells in ventral roots express high levels of glial cell line-derived neurotrophic factor (GDNF) (Hoke et al., 2006; Brushart et al., 2013). This difference in growth factor expression has been proposed to be the mechanism through which preferential motor reinnervation occurs, with DRG neurons choosing paths with high NGF, and motoneurons entering paths with high GDNF. With no treatment, in rodents, neurotrophin expression peaks 15 days after injury and declines back to baseline by day 30. The day 15 peak coincides with the onset of pathway preference for regenerating axons (Gordon, 2015). ES, however, dramatically increases NGF secretion from Schwann cells for 3 days following stimulation (Koppes et al., 2014), possibly providing an earlier signal to regenerating sensory axons as to which pathways to take, and thus improving pathway targeting.

### Exercise Treatment

For years, the evidence for exercise enhancing regeneration was not as clear as the evidence for ES. Many different types of exercise with varying intensities applied at different times prior to or after injury have resulted in conflicting results. It was hypothesized that increased neuronal activity through exercise would enhance regeneration as early as 1979, but early studies utilizing treadmill training, voluntary wheel running, and swimming found unfavorable results (Hoffer et al., 1979; Herbison et al., 1980a; Gardiner et al., 1984; Badke et al., 1989; van Meeteren et al., 1998; Tam et al., 2001). These experiments largely focused on the effect of exercise on muscle fiber alterations and muscle function, and did not probe the effect of exercise on axon regeneration.

The change in emphasis from the effect of exercise on denervated muscle to the effect of exercise on injured spinal motoneurons and DRG neurons encouraged scientists to continue researching exercise, despite previous underwhelming results. In 2008, English and colleagues tested the efficacy of interval training (short high-speed sprints followed by periods of rest) in enhancing regeneration as a model that resembles how mice voluntarily run (De Bono et al., 2006; Sabatier et al., 2008). They found the surprising result that this regimen was effective only in female mice, and in fact the more commonly used training regimen of slow continuous treadmill walking was effective only in males (Wood et al., 2012). This previously unknown sex difference could have affected outcomes in numerous exercise experiments. For example, Seo et al. treated intact male rats with either high or low intensity treadmill training before culturing their DRGs and found only low intensity treadmill training increased neurite outgrowth (Seo et al., 2009). Their treadmill training regimen was very similar to the one used by Wood et al. that proved effective only in male mice, and the results of this experiment could have been different had females been included. Many of the prior experiments mentioned used animals of only one sex, and this could explain some of the variability in the effects of exercise (Tables 2–4).

There are a few advantages to exercise over ES. For example, while ES may increase misdirection of motoneurons reaching target muscles, treadmill training enhances motoneuron regeneration without decreasing topographic specificity (English et al., 2009). The mechanism of ES is to accelerate crossing the site of injury by regenerative sprouts; exercise does the same but also sustains pro-growth signaling throughout the process of regeneration (Gordon and English, 2016). There is also evidence that the enhancing effects of 2 weeks of exercise are more robust than that of a single bout of ES (Sabatier et al., 2008; Wood et al., 2012; Gordon and English, 2016). In 2009; Asensio-Pinilla et al. combined treadmill training with a single bout of ES given at the time of injury, and found greater enhancement of muscle reinnervation in the initial phase of recovery compared to either treatment alone (Asensio-Pinilla et al., 2009). Thus, after inauspicious beginnings, exercise has shown great promise as a treatment in the field of peripheral nerve regeneration.

### Optogenetic Stimulation

The advent of optogenetics enabled cell-specific neuronal activation with the use of the light-sensitive cation channel, Channelrhodopsin (ChR2) (Krook-Magnuson et al., 2014). Whereas ES stimulates all cells within the nerve (including Schwann cells and various immune cells) and exercise likely affects cells throughout the entire body, specific neuronal activation can be achieved using optogenetics by expressing ChR2 only in neurons. Park et al. were the first to demonstrate the efficacy of light stimulation in enhancing regeneration by replicating the common ES protocol using light stimulation of 20 Hz for 1 h on explanted neonatal DRGs (Park et al., 2015). Although they tested a number of different stimulation regimens, the 1 h of 20 Hz stimulation provided the largest effect on neurite outgrowth. Ward et al. recapitulated this *in vivo*, finding that 1 h of 20 Hz light stimulation of light-sensitive neurons enhanced axon regeneration only in the light-sensitive cells (Ward et al., 2016, 2018).

## Mechanisms

### Neurotrophins

Activity-dependent treatments require neuronal neurotrophin production. ES increases neurotrophin expression in Schwann cells, DRG neurons, and motoneurons (Al Majed et al., 2000a; English et al., 2007; Wan et al., 2010; Koppes et al., 2014). Electrically stimulating Schwann cells increases their secretion of NGF specifically, and not BDNF (Koppes et al., 2014). While Schwann cell NGF is sufficient to promote axon growth, the study of axon regeneration through nerve grafts made acellular by repeated freezing and thawing has demonstrated that stimulation of Schwann cells (and other cell types) is not required for the efficacy of ES to enhance axon regeneration (English et al., 2007; Koppes et al., 2014). Moreover, the use of optogenetics to stimulate neurons selectively has shown that specific neuronal activation is sufficient to enhance regeneration (Ward et al., 2016). ES is also effective in promoting regeneration in nerves that have been repaired months after injury, when Schwann cells have stopped secreting neurotrophins and have started to die off (Sulaiman and Gordon, 2000; Hoke et al., 2006; Brushart et al., 2013; Huang et al., 2013; Elzinga et al., 2015). Thus, while activity-dependent treatments may increase Schwann cell neurotrophin secretion, this is not required for their enhancing effects.

Unlike non-neuronal cells, *neuronal* neurotrophin secretion *is* required for the efficacy of activity-dependent treatments. Genetically deleting NT4/5 or BDNF from Schwann cells does not alter the efficacy of ES or treadmill training in enhancing axon growth, but deleting these neurotrophins from neurons abolishes the effectiveness of these activity-dependent treatments (English et al., 2007; Wilhelm et al., 2012). Both exercise and ES have been shown to increase neuronal BDNF and its receptor, trkB (Al Majed et al., 2000a; Gomez-Pinilla et al., 2002; English et al., 2007; Park and Höke, 2014; Park et al., 2015). Through co-culturing light-sensitive DRG explants with wild type DRGs, Park et al. demonstrated that the BDNF secreted in response to light stimulation was sufficient to increase neurite outgrowth not only from cells in a light-sensitive (ChR2-expressing) DRG, but also in neighboring ganglia derived from wild type mice. Protein analysis of the media revealed increased BDNF and NGF secretion in response to optical stimulation from the light-sensitive DRGs only (Park et al., 2015).

There is a dose-dependence in activity-dependent treatments for enhancing nerve regeneration. Whereas 1 h of 20 Hz stimulation has been shown to enhance DRG regeneration after injury, an increase to just 3 h of ES decreased sensory neuron regeneration, and was associated with a downregulation in expression of the regeneration associated gene, GAP-43 (Geremia et al., 2007). *In vitro*, neurites from DRG explants containing ChR2 had higher rates of growth with stimulation paradigms resulting in 72k pulses of light (1 h 20 Hz, 2 h 10 Hz, 4 h 5 Hz) than stimulation paradigms that resulted in a higher number of pulses (20 Hz for 1–3 days) or much lower number of pulses (20 Hz for 15 min) (Park et al., 2015). Three days of continuous depolarization through ES or high concentrations of KCl results in complete failure of dissociated DRG neurons to grow neurites in culture (Enes et al., 2010). For motoneurons, high intensity exercise or repeated bouts of ES result in decreased sprouting and fewer synaptic contacts at neuromuscular junctions (Tam et al., 2001). Application of 1 h of 20 Hz ES every third day for 2 weeks after sciatic nerve transection and repair did not enhance the regeneration of motor axons in mice (Park et al., under review). Interestingly, exogenous application of BDNF resulted in a dose-dependent enhancement of axon regeneration as well (Boyd and Gordon, 2002). Low to modest doses produced enhanced axon regeneration, but higher doses inhibited regeneration. Treatments with high doses of BDNF caused p75^NTR^ activation, which prevented DRG neurite outgrowth (Boyd and Gordon, 2002).

### Neuronal Activity

The success of activity-dependent treatments in promoting axon regeneration requires activation of the injured neurons. Treating the neurons proximal to the stimulation site with tetrodotoxin (TTX) to block their ability to conduct antidromic action potentials abolishes the effect of ES, despite the continued orthodromic firing of distal axons and muscle fibers (Al Majed et al., 2000b). Similarly, inhibition of motoneuron activity during treadmill training, using bioluminescent optogenetics (BL-OG), abolishes the enhancing effect of exercise on motoneuron regeneration (Jaiswal et al., 2017). Whether the increased activation needed to promote axon regeneration requires action potential generation is not entirely clear. Enhancement of regeneration of axons of many more motoneurons than are likely to be brought into full activity is found after treatments with exercise at a slow treadmill speed (Gordon and English, 2016). Simply increasing the excitability of injured neurons using chemogenetics could be sufficient to enhance regeneration (Jaiswal et al., 2018).

Although Park et al. found that BDNF secretion from neighbors can stimulate regeneration in neurons that were not activated *in vitro*, optogenetic stimulation *in vivo* of only motoneuron axons did not enhance DRG axon regeneration, nor vice versa (Ward et al., 2018). When BDNF is knocked out in only a subset of neurons, those specific neurons do not benefit from exercise treatment (Wilhelm et al., 2012). Thus, it appears neuronal BDNF is acting as an autocrine signal facilitating enhanced regeneration (English et al., 2014; Gordon, 2016).

### Androgens

The sex difference found in response to different exercise regimens led to the hypothesis that androgen receptor signaling was involved in activity-dependent treatments. The effect of androgens in enhancing peripheral nerve regeneration had already been thoroughly explored several years prior (Fargo et al., 2009). All motoneurons contain androgen receptors, though testosterone is not required for spontaneous regeneration—treating animals of both sexes with the androgen receptor blocker flutamide does not inhibit regeneration, nor does castration of males (Freeman et al., 1995; Thompson et al., 2013). Application of exogenous androgens in males and females, however, enhances axon regeneration in both cranial and spinal nerve injuries (Kujawa et al., 1989, 1991; Jones, 1993; Freeman et al., 1995; Tanzer and Jones, 1997; Brown et al., 1999). This effect is androgen receptor-dependent, and blocking the androgen receptor with flutamide prevents testosterone-induced enhanced regeneration (Kujawa et al., 1995). In females, treating mice with anastrazole, an aromatase inhibitor which blocks the conversion of testosterone into estradiol, also dramatically enhanced axon regeneration, without increasing serum androgen levels (Thompson et al., 2013).

The sex difference in response to exercise regimens was evidence that androgen receptor signaling and activity-dependent treatments were linked. Slow, continuous treadmill training resulted in an increase in serum testosterone levels in males, though no similar increase was found for females with interval training (Wood et al., 2012). Castrating males prior to treadmill training abolishes its enhancing effect, which cannot be rescued with interval training (Wood et al., 2012). Treating both sexes with flutamide before appropriate exercise regimens abolishes the effectiveness of this treatment in enhancing peripheral nerve regeneration (Thompson et al., 2013). Testosterone is also necessary for the beneficial effects of ES—castrated rats treated with ES have poorer regeneration compared to littermates who are treated with exogenous testosterone (Hetzler et al., 2008; Sharma et al., 2009). As with exercise, flutamide blocks the enhancing effects of ES in both males and females (Thompson et al., 2013). Conversely, combined exogenous androgen treatment with ES enhances facial nerve regeneration in gonadally intact rats (Sharma et al., 2010b).

Androgens regulate BDNF and its receptor, trkB, in motoneurons (Osborne et al., 2007; Ottem et al., 2010; Sharma et al., 2010a; Verhovshek et al., 2010). Exercise elicits an upregulation of testosterone that is sustained and could result in an increased duration of BDNF and trkB expression (Thompson et al., 2013; English et al., 2014). ES elicits an early increase in BDNF expression, whereas exogenous androgen application results in a later and longer-duration increase in expression. Combining the two treatments results in an additive upregulation of BDNF and could explain the improved recovery over either treatment alone (Sharma et al., 2010a).

## Synaptic Rearrangements

After peripheral nerve injury both excitatory and inhibitory synaptic inputs onto injured motoneurons are withdrawn (Blinzinger and Kreutzberg, 1968; Brannstrom and Kellerth, 1998; Linda et al., 2000; Oliveira et al., 2004). If motor axons regenerate and reinnervate muscle targets, many of these inputs are restored but, for those expressing the vesicular glutamate transporter 1 (VGLUT1) and arising from muscle spindles, a gradual withdrawal of their central axonal processes from the ventral horn follows, resulting in a permanent loss of these synaptic inputs (Alvarez et al., 2011; Rotterman et al., 2014). In animals treated with exercise during the first few days following sciatic nerve transection and repair, the extent of synaptic contacts between these important sources of proprioceptive feedback and motoneurons is not reduced (English et al., 2011; Liu et al., 2014; Krakowiak et al., 2015). This robust connectivity by VGLUT1+ inputs is retained at least 12 weeks later. No similar effect is found if the onset of the exercise treatment is delayed (Brandt et al., 2015). Application of 1 h of 20 Hz ES had no effect on synaptic coverage after nerve injury, but repeated applications every third day for 2 weeks resulted in an effect similar to that observed using exercise (Park et al., under review). It is not clear whether this effect of these activity-dependent therapies is the prevention of the original synaptic withdrawal, a stimulation of new synapse formation to replace the withdrawn inputs, or some combination of both. More studies are needed.

It is clear that BDNF plays a role in maintaining and preserving synaptic inputs on motoneurons. Without exercise, axotomized motoneurons lose approximately 35% of their overall synaptic coverage (Krakowiak et al., 2015). This effect is BDNF-dependent—knocking out BDNF in a subset of motoneurons reduces synaptic coverage in those specific cells in intact animals, and this synapse loss cannot be rescued with exercise (Krakowiak et al., 2015). Wild-type motoneurons within an animal maintain their synaptic contacts after nerve injury with exercise, but those in which BDNF has been knocked out do not (Krakowiak et al., 2015).

## BDNF Val66Met Polymorphism

Given the relationship between activity-dependent treatments and BDNF, any genetic mutations altering BDNF signaling among the human population could affect the success of these treatments. Such a mutation exists—a common single nucleotide polymorphism in the *BDNF* gene. The G to A mutation at site 196 results in a Valine to Methionine substitution in the 66th codon (Figure 1). This polymorphism was first described by Egan et al. in 2003 and was quickly identified as incredibly common—the met allele of the *BDNF* gene is present in 25% of the American population and up to 50% of East Asian populations (Egan et al., 2003; Shimizu et al., 2004). Carrying the met allele was originally described as a risk factor for schizophrenia (Egan et al., 2003). It has since been linked to numerous other disorders and diseases, including Alzheimer's disease, obsessive compulsive disorder, anorexia nervosa, and bipolar disorder (Neves-Pereira et al., 2002; Sklar et al., 2002; Egan et al., 2003; Hall et al., 2003; Ribases et al., 2003; Notaras et al., 2015). Physiologically, Met-carriers have been found to have decreased hippocampal volume, and cells transfected with the Met allele have altered activity-dependent secretion of BDNF (Egan et al., 2003).

Testing for deficient activity-dependent secretion of BDNF in humans can be tricky. Generally, BDNF secretion is measured through serum as an indirect measure of neuronal BDNF, and exercise is a reliable method to increase serum BDNF levels (Berchtold et al., 2005; Elfving et al., 2010; Klein et al., 2011; Szuhany et al., 2015). Although one study has found that healthy adult Met-carriers did have increased serum BDNF after exercise (Helm et al., 2017), others have found serum levels of BDNF did not increase after high intensity exercise in elderly (Nascimento et al., 2015), spinal cord injured (Leech and Hornby, 2017), or healthy Met-carriers (Lemos et al., 2015). In mice expressing the met allele, exercise results in deficient mRNA production as well as decreased protein expression of BDNF (Ieraci et al., 2016). These deficiencies in exercise-induced BDNF secretion mirror the findings in cultured neurons expressing the Met allele, and the use of cells transfected with the met allele *in vitro* as well as the development of a transgenic mouse have allowed researchers to elucidate the mechanism behind this deficient secretion (Egan et al., 2003; Chen et al., 2004, 2006).

The valine to methionine substitution in this SNP occurs in the prodomain of the BDNF protein (Egan et al., 2003). Although it does not affect the ability of mBDNF to bind its receptor, this substitution results in disorganized folding of the prodomain, resulting in abnormal interactions with sortilin (see above) (Chen et al., 2004; Anastasia et al., 2013). BDNF_Met_ is thus packaged inefficiently into calcium-sensitive secretory vesicles, accounting for the deficient activity-dependent secretion that has been reported (Chen et al., 2004). Being heterozygous for the met allele does not protect from this deficient BDNF secretion—BDNF forms homodimers, and in cells heterozygous for the met allele, BDNF_Met_ dimerizes with BDNF_Val_ and prevents its packaging into Ca^2+^-regulated secretory vesicles (Kolbeck et al., 1994; Chen et al., 2004). Analysis of activity-induced BDNF secretion from cultured hippocampal cells bears this out—those cells heterozygous for the Met allele have deficient activity-dependent secretion despite the presence of one copy of the BDNF_Val_ allele (Chen et al., 2006). Furthermore, once secreted, BDNF availability may be affected by binding with the cleaved prodomain. The prodomain binds BDNF with high affinity, and the met allele results in enhanced BDNF binding and slower dissociation once bound (Uegaki et al., 2017). This could limit the availability of BDNF to bind its receptors.

Activity-dependent secretion of BDNF relies not only on packaging into calcium-sensitive vesicles, but also on the spatial targeting of mRNA into distal processes where BDNF can be locally translated (Chiaruttini et al., 2008). This targeting is achieved through binding of BDNF mRNA with translin, a DNA/RNA binding protein involved in dendritic trafficking of mRNAs (Li et al., 2008). The G to A mutation at site 196 disrupts translin binding of BDNF mRNA, and thus Met-carriers have deficient trafficking of BDNF mRNA to distal processes (Chiaruttini et al., 2009). Moreover, the transcripts containing exon VI, which is upregulated by exercise, and exon IV, which is calcium-sensitive, are found in reduced levels in the hippocampus of mice homozygous for the met allele (Tao et al., 1998; Baj et al., 2012; Mallei et al., 2015). These transcripts, along with those containing exon II, are generally trafficked to distal processes (Baj et al., 2011).

In addition to deficient activity-dependent BDNF secretion, the met allele may result in increased p75^NTR^ activation. Unlike BDNF_Val_, when the prodomain is cleaved from BDNF_Met_, it is bioactive and able to activate p75^NTR^ with the help of SorCS2, a member of the sortilin family of receptors (Deinhardt et al., 2011; Anastasia et al., 2013). *In vitro*, application of exogenous prodomain protein results in growth cone collapse and dendritic spine disassembly (Anastasia et al., 2013; Giza et al., 2018). Stimulating cells with high KCl concentration results in activity-dependent secretion of both Val and Met prodomains, though secretion is deficient in Met-carriers (Anastasia et al., 2013). Although endogenous secretion of the Met prodomain has yet to be linked to alterations in dendrites, decreased arborization has been found in hippocampal and cortical neurons (Chen et al., 2006; Liu et al., 2012).

The deficit in activity-dependent release of BDNF led to the hypothesis that activity-dependent treatments to enhance axon regeneration after peripheral nerve injury would be ineffective in this population. Using a mouse model of this polymorphism which recapitulates certain phenotypic aspects of the human population such as decreased hippocampal volume and increased anxiety-like behavior (Chen et al., 2006), we tested the efficacy of treadmill training on motor axon regeneration 4 weeks after complete sciatic nerve transection and repair in mice both heterozygous and homozygous for the met allele of the *Bdnf* gene (McGregor et al., under review). Exercise was completely ineffective in enhancing axon regeneration in the Met-carriers. However, peripheral axon regeneration in Met-carriers was surprisingly enhanced without any treatment (McGregor et al., under review). One possibility for the failure of exercise to enhance regeneration in Met-carriers is a ceiling effect—exercise was not able to further enhance an already accelerated regeneration. The question of *why* regeneration is accelerated remains to be explored. The enhanced regeneration was found both *in vivo* and in cultured DRG neurons, indicating that enhanced axon outgrowth is a neuronal trait in Met-carriers. This unanticipated result is some of the first good news regarding what is a maligned SNP, although others have reported the met allele may also be protective in stroke and traumatic brain injury (Krueger et al., 2011; Rostami et al., 2011; Qin et al., 2014; Failla et al., 2015). Thus, in human populations there may be striking differences in response to peripheral nerve injury dependent on individual gene expression. Enhanced regeneration associated with the Val66Met polymorphism may explain the persistence of the mutation within the human population.

## Conclusion

The suboptimal regeneration of peripheral nerves presents a challenge in medical care. Neurotrophins, particularly BDNF, have been studied for their pro-growth properties, and treatments that stimulate endogenous release of neurotrophins have been successful in enhancing regeneration in animal models. These treatments are currently being tested to enhance peripheral nerve regeneration in patients with some success (Gordon et al., 2010; Wong et al., 2015). The existence of genetic polymorphisms in the *bdnf* gene, however, will affect the outcome of these experiments, and preliminary investigations as to the efficacy of activity-dependent treatments in individuals with the met allele will hopefully spur the field toward personalized medicine. Activity-dependent treatments can be a powerful tool for those responsive to them, and for the rest, new therapies that do not rely on endogenous BDNF-signaling must be developed.

## Author Contributions

All authors listed have made a substantial, direct and intellectual contribution to the work, and approved it for publication.

### Conflict of Interest Statement

The authors declare that the research was conducted in the absence of any commercial or financial relationships that could be construed as a potential conflict of interest.
